# Assessing Weight Stigma: Validating Attitudes and Beliefs Questionnaires Among Future Healthcare Professionals

**DOI:** 10.7759/cureus.66345

**Published:** 2024-08-07

**Authors:** Laura Mihalache, Alina Delia Popa, Andreea Gherasim, Otilia Nita, Mariana Graur, Oana Madalina Rosu, Lidia Iuliana Arhire

**Affiliations:** 1 Internal Medicine II, University of Medicine and Pharmacy “Grigore T. Popa” Iasi, Iasi, ROU; 2 Internal Medicine II/Nursing, University of Medicine and Pharmacy “Grigore T. Popa” Iasi, Iasi, ROU; 3 Internal Medicine, University “Ștefan cel Mare” of Suceava, Suceava, ROU

**Keywords:** nutrition, validity, stigma, obesity, nursing students

## Abstract

Background

This study aimed to establish the reliability and validity of the Anti-Fat Attitudes (AFA) questionnaire and the Beliefs About Obese Persons (BAOP) scale.

Methodology

A convenience sample of 257 students from three distinct sections of the University of Medicine and Pharmacy “Grigore T. Popa” Iasi (Medical Faculty, Nutrition and Dietetics, and Nursing) participated in an observational study. Construction validity was tested with exploratory factor analysis. The students completed a form containing sociodemographic data, the AFA questionnaire, and the BAOP scale. Weight and height were self-reported and used to determine the body mass index.

Results

The value of the Cronbach alpha coefficient for the AFA questionnaire indicated adequate internal consistency (0.862). The exploratory factor analysis identified the following three factors corresponding to the original questionnaire: Dislike, Fear of Fat, and Willpower. We validated a single-factor structure of the BAOP scale, which had adequate internal consistency (0.781). There were statistically significant differences (AFA: p = 0.02; BAOP: p = 0.03) between the scores of the students from Nutrition and Dietetics, Nursing, and General Medicine.

Conclusions

This study demonstrated that the AFA questionnaire and the BAOP scale could be used to evaluate weight stigma in healthcare students, providing a useful tool to assess the effects of weight stigma awareness interventions in this population.

## Introduction

Obesity affects more than one billion people worldwide and is associated with important cardiovascular and metabolic comorbidities. The obesity epidemic was related to the increasing number of cases of type 2 diabetes in the last decades. A large epidemiological study showed a high prevalence of obesity (31.90%) and abdominal obesity (73.90%) in the adult population in Romania [1]. The increasing number of patients with obesity leads to a higher number of referrals of these patients for health problems. Along with sexism and racism, stigma related to weight is one of the most prevalent forms of discrimination in contemporary societies [2]. Weight discrimination is the last manifestation of socially permissible stigma, according to Kilbourne [3]. It is estimated that 19-42% of adults experience it [4].

The obesity stigma consists of society’s devaluation and stereotypes, including the beliefs that people with obesity are indolent, unintelligent, without self-control, worthless, negligent, non-adherent to treatment, and lacking self-discipline or determination. Weight bias, either explicit (conscious and overt) or implicit (unconscious and subliminal) [2], represents negative beliefs, attitudes, judgments, and assumptions about individuals living with overweight or obesity [5]. Overweight individuals face disparaging remarks and misbehavior in various settings. They may seek treatment for both weight-related and unrelated requirements, and healthcare professionals’ weight bias may worsen health concerns [6], which often leads to delaying medical appointments, and avoidance of preventative or continuing care, thus contributing to worse health outcomes and quality of life [7]. According to a World Health Organization report [2], nearly 70% of adults with obesity have experienced stigma from healthcare professionals. Two-thirds of participants with obesity in each nation reported stigmatizing experiences with health professionals, according to a multinational study conducted in six nations on three continents [8]. Despite the best intentions of healthcare professionals to provide quality care, these negative attitudes can reduce the quality of care given to patients with obesity. There is evidence that healthcare professionals have a weight bias toward individuals who are overweight or obese, even when the presenting concerns are unrelated to obesity [9] by offering unsolicited advice, prescribing different treatments, and displaying negative facial expressions [10]. As a result, individuals with obesity tend to experience tension related to healthcare resulting in negative health outcomes [8,11]. Contrary to recent evidence indicating that obesity is a complex chronic problem [12,13], these negative attitudes on the part of health professionals are closely related to the belief that obesity is primarily a matter of individual responsibility.

Obesity is a visible trait, and individuals with extreme degrees of obesity experience greater stigmatization. Both nurses and nursing students had a negative perception of obesity and were unlikely to attribute positive characteristics to people with obesity [14]. Being subjected to weight stigma leads to heightened activation in brain regions responsible for regulating hunger, specifically the dorsal striatum and thalamus. This activation is particularly manifested in overweight individuals exposed to high-calorie foods [15]. Furthermore, the existence of both explicit and implicit weight bias among students at various medical universities in Australia [16] indicated the necessity of designing approaches to address social stigma. Providing targeted education and raising awareness among medical students regarding the detrimental consequences of stigmatizing individuals who are overweight holds the potential to enhance the level of care for these individuals [17,18].

Healthcare university students will have an increasing number of patients with obesity. The lack of awareness of obesity bias and its effects would jeopardize their future relationships with patients and the standard of care. Understanding the complexity of obesity and its complications, as well as the weight stigma and its detrimental effects, will help reduce disparities in future patients’ access to treatment without bias [16,19].

Previous research concluded that despite numerous studies examining weight bias, the extant literature failed to capture the full image of the obesity stigma that is present among healthcare students [20]. Most weight stigma validation studies were not conducted on students pursuing healthcare degrees [21]. Moreover, regardless of growing global interest in studies on anti-fat attitudes, the psychometric characteristics of the Anti-Fat Attitudes (AFA) questionnaire have rarely been studied in non-English-speaking populations [20,21].

To our knowledge, in Romania, there are no studies investigating weight bias among healthcare students and rising awareness of weight stigma could be important in the development of destigmatization strategies [22]. In this study, we aimed to address this gap by validating two questionnaires that assess obesity bias in healthcare students, namely, the Beliefs About Obese Persons scale [20] and the AFA questionnaire [23].

## Materials and methods

Study design

We conducted a cross-sectional study on a convenience sample of students preparing for healthcare professions to examine the psychometric properties of two questionnaires that assess stigma in patients with obesity, i.e., the BAOP scale [21] and the AFA questionnaire [23].

Participants and data collection

The participants were recruited from the students at the University of Medicine and Pharmacy “Grigore T. Popa” Iași, which is the largest in the northeastern part of the country. The questionnaire was addressed only to students from the following specializations: Nutrition and Dietetics, Medicine, and Nursing. We invited students to complete the form containing the weight stigma questionnaires between February 15 and April 15, 2024. After being briefed on the objective and scope of the study, students who declined to participate were excluded from data collection. The AFA questionnaire contains 13 questions, while the BAOP scale has only eight. We estimated there would be 130 participants based on the recommendation to conduct exploratory analysis on samples at least 5-10 times the number of items in the questionnaire [24]. The sample size estimation was performed with the G*Power 3.1.9.7 program. We conducted a power analysis for an analysis of variance test with fixed effects because we compared the mean differences in the scores between three groups of participants. The sample size was estimated to be 269 participants to detect a medium effect size of 0.25 with a power of 0.80 at a 0.05 significance level.

The inclusion criteria were normal-weight students with an age greater than 18 years old, affiliation with the University of Medicine and Pharmacy “Grigore T. Popa” Iași, and the presence of a signed consent form.

Ethics considerations

The investigation was conducted with the authorization of the University of Medicine and Pharmacy “Grigore T. Popa” Ethics Committee (approval number: 391/30.01.2024). The data were anonymized to protect the privacy of participants.

Measurements

Demographic data collected included age, sex, and the faculty attended (Medicine, Nursing and Nutrition, and Dietetics).

The AFA questionnaire is a 13-item Likert scale structured into the following three sections: Dislike, Fear of Fat, and Willpower [25]. The first section, Dislike, consists of seven items that assess the negative feelings associated with obesity. The Fear of Fat section includes three items related to worries about weight gain. The final section, Willpower, contains three items addressing the fact that individuals with obesity have a weak desire to lose weight. Each item can receive a score between 0 (intense disagreement) and 9 (complete agreement). The total score ranges between 0 and 117, with a higher score representing a stronger negative attitude toward patients with obesity.

The BAOP scale consists of eight items rated on a six-point Likert scale (-3 = firmly disagree to 3 = strongly agree). The total score is the sum of the points obtained on the scale after six negatively worded items are reversed and 24 is added to the sum of the eight-item scores. The score can range between 0 and 24. A higher score indicates firmer beliefs that individuals with obesity cannot control their weight [26].

Questionnaire validation

Translation Process

Two independent translators fluent in both English and Romanian translated the questionnaires from the original language (English) to Romanian. A committee of experts, consisting of two additional translators and three nutrition specialists, assessed the translations and integrated them into a single version.

Content Validity

The committee of experts compared the back-translated questionnaire to the original to identify any discrepancies or issues requiring attention. Two specialists evaluated the intelligibility, sufficiency, and significance of the translated questionnaires concerning the appropriate assessment of obesity-related beliefs and attitudes.

Face Validity

The AFA and BAOP questionnaire items were pilot-tested for accessibility and ambiguity on six students (two from the Medicine Faculty, two from the Nursing Faculty, and two from the Nutrition and Dietetics Faculty) who were not included in the final survey. If a question was ambiguous, participants were asked for their feedback. No changes were made in concluding this procedure.

Psychometric evaluation

We determined the mean and standard deviation (SD) for each item. By correlating each element’s score with the total scale score, the capacity of each item to discriminate between individuals with differing levels of attitudes or beliefs was determined. All items with a significant correlation (r) higher than 0.3 were retained [26].

Construct Validity

Identifying latent components/factors via exploratory factorial analysis was the next step. The Kaiser-Meyer-Olkin (KMO) sampling adequacy measure was used to indicate if the sample size was sufficient for a reliable factor extraction [27]. Bartlett’s sphericity test was applied to determine the presence of multicollinearity, and the p-value of Bartlett’s test of sphericity was regarded as significant if it was less than 0.05. Individual factor loading values are required to be larger than 0.25. We regarded variables that contributed to the construction of the factor as having a correlation coefficient greater than 0.40.

Reliability

The Cronbach’s alpha coefficient was employed to evaluate the internal consistency of both AFA and BAOP [28], with values above 0.7 considered acceptable.

Statistical analysis

Survey responses were included in a database in Microsoft Office Excel 2007 (Microsoft Corp., Redmond, WA, USA). The R Studio (version 1.4.1106) packages “psych,” “factoextra,” and “GPArotation” were used for statistical processing. The Kolmogorov-Smirnov test was applied to examine the normality of the questionnaire scores’ distribution. Due to the absence of normal distribution of the scores on the two questionnaires, the differences between the groups were determined using the Kruskal-Wallis test.

## Results

Participants’ characteristics

The final sample included 257 people (232 women and 25 men) out of 4,127 enrolled students (3,176 women and 951 men), with an age range of 18 to 50 years. Regarding the age distribution, 57.97% (149 individuals) of the respondents were between 18 and 25 years old, 12.45% (32 individuals) were over 25 years old, and 29.56% (72 students) were between 30 and 50 years old. There were 45 (17.51%) respondents from the Medicine Faculty, 98 (38.13%) respondents from the Nursing Faculty, and 114 (44.35%) respondents from the Nutrition and Dietetics Faculty. All participants had a normal body mass index (BMI).

Item analysis

The mean AFA score was 35.81, with an SD of 21.66. The median score was 33, ranging between 0 to 101 (interquartile range (IQR) = 34): 25% of the participants scored below 18, while 25% of the students scored above 52. The mean scores of the three subscales were as follows: Dislike (11.1 ± 10.9), Fear of Fat (13.4 ± 8.89), and Willpower (11.3 ± 7.83).

**Figure 1:**
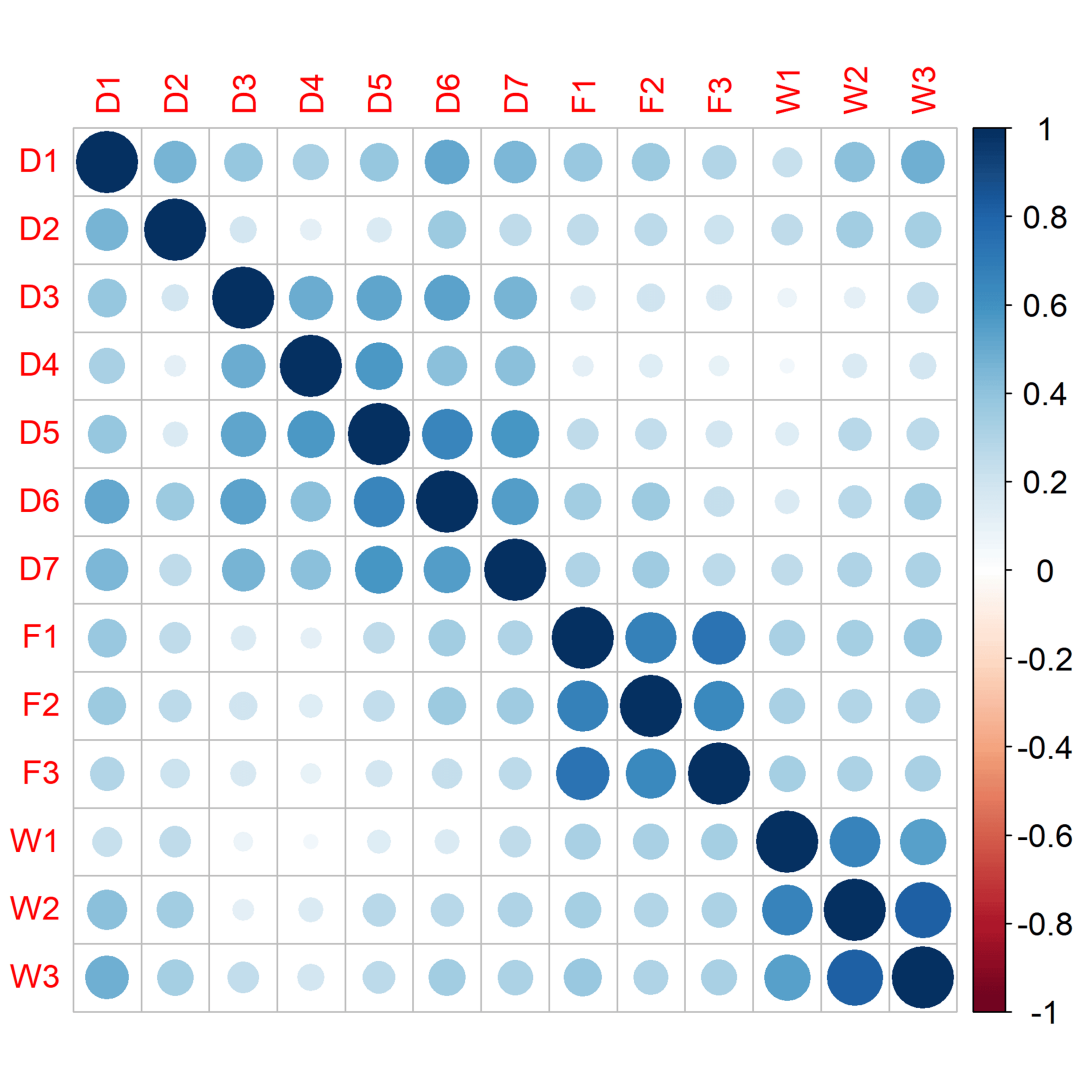
Anti-Fat Attitudes questionnaire: items correlation matrix.

**Figure 2:**
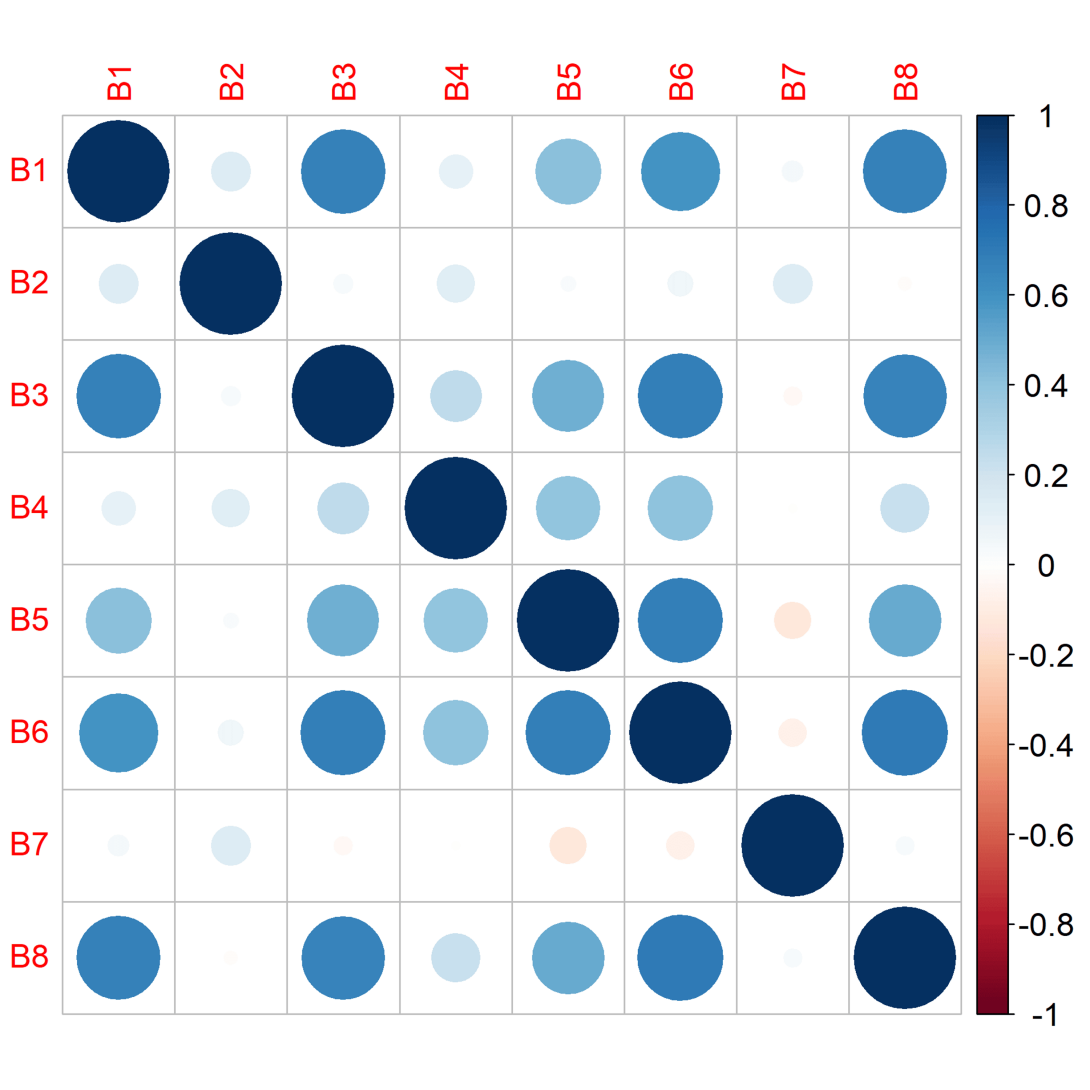
Beliefs About Obese Persons scale: items correlation matrix.

The BAOP scale scores ranged from 0 to 24, the mean score was 18.5 (SD = 4.67; IQR = 5), and the median score was 20. The lowest 25% of ratings were below 17, while the highest 25% were above 22. The correlation coefficients with the overall score were greater than 0.3 in both the AFA and BAOP questionnaires, apart from two questions from the BAOP scale (B2: “Obesity is frequently the result of a biological disorder” and B7: “Obesity is rarely the result of a lack of willpower”) (Table 1, Figures 1, 2). However, we kept these items in our analysis because we wanted to preserve the original form of the BAOP scale. Furthermore, other authors considered an r value of 0.2 to be a threshold for the item discrimination analysis [29].

**Table 1:** Results of the item analysis. **: p < 0.001. AFA questionnaire: D1-D7 - items from the Dislike section; W1-W3 - items from the Willpower section; F1-F3 - items from the Fear section. B1-B8 - items of the BAOP questionnaire. AFA = Anti-Fat Attitudes; BAOP = Beliefs About Obese Persons

Item	Mean	Standard deviation	Item total correlation
AFA			
Total score	35.813	21.66	—
D1	2.039	2.6	0.668^**^
D2	4.393	3.2	0.559^**^
D3	0.638	1.66	0.501^**^
D4	0.681	1.79	0.441^**^
D4	0.802	1.86	0.535^**^
D6	1.315	2.29	0.628^**^
D7	1.257	2.16	0.587^**^
F1	3.829	3.25	0.719^**^
F2	5.16	3.32	0.708^**^
F3	4.428	3.44	0.667^**^
W1	4.37	3.07	0.609^**^
W2	3.716	2.95	0.705^**^
W3	3.187	2.82	0.716^**^
BAOP			
Total score	18.5253	4.67	—
B1	0.1323	2.11	0.528^**^
B2	1.0934	1.63	0.238^**^
B3	0.0467	2.02	0.557^**^
B4	-0.393	1.71	0.612^**^
B5	-0.0817	1.94	0.653^**^
B6	0.1556	2.08	0.67^**^
B7	-0.4125	1.68	0.215^**^
B8	0.2724	2.28	0.535^**^

Construct validity

**Figure 3:**
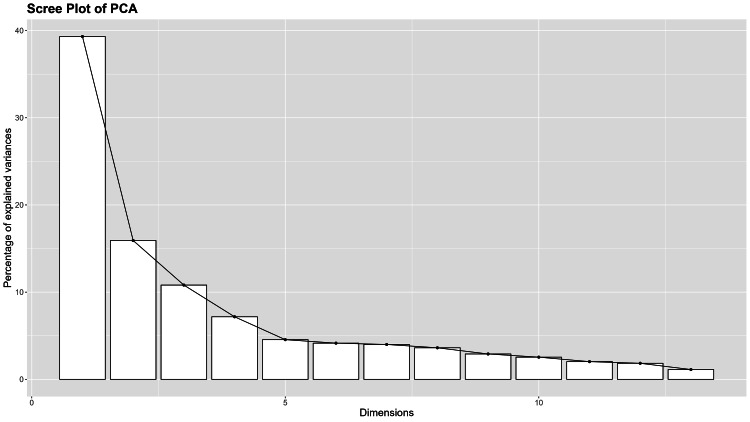
Anti-Fat Attitudes questionnaire: scree plot.

The KMO value of the AFA scale was 0.834. The significance level of Bartlett’s sphericity test was less than 0.001, indicating that factor analysis of the data could be utilized. The exploratory factorial analysis for the detection of latent components revealed three factors that collectively accounted for 66% of the total variance. The extracted factors were similar to the original AFA questionnaire: Dislike (25.8% of variance), Fear of Fat (21.3% of variance), and Willpower (18.9% of variance) (Table 2, Figure 3). A scree plot was used to determine the number of factors (components) to be retained in the analysis. It shows the eigenvalues associated with each component in descending order. The x-axis (dimensions) represents the number of factors and the y-axis represents the eigenvalue size. On the scree plot, we observed a clear elbow at the third component, suggesting that the first three components (Dislike, Fear of Fat, and Willpower) explained most of the variance and the additional components only contributed in a small proportion.

**Table 2:** Anti-Fat Attitudes questionnaire: rotated component matrix.

Item	Dislike	Fear of Fat	Willpower
D1	0.524	0.244	0.428
D2	0.461	0.173	0.460
D3	0.77	0.060	0.041
D4	0.748	-0.031	0.029
D4	0.821	0.096	0.118
D6	0.769	0.225	0.189
D7	0.696	0.222	0.218
F1	0.142	0.864	0.223
F2	0.193	0.828	0.179
F3	0.076	0.863	0.195
W1	-0.024	0.2151	0.768
W2	0.119	0.1224	0.912
W3	0.201	0.1451	0.854

**Figure 4:**
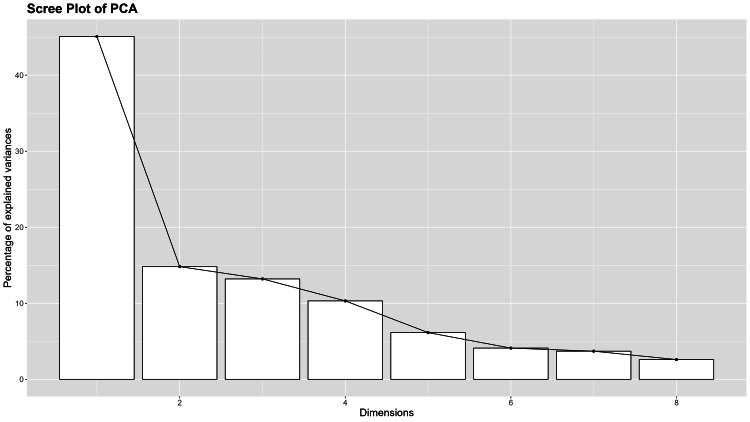
Beliefs About Obese Persons scale: scree plot.

The BAOP scale had a KMO value of 0.817; and the significance level of Bartlett’s sphericity test was below 0.001 (Table 3, Figure 4). The exploratory analysis isolated a structure with a single factor explaining 45.08% of the variance. Two items were not included in this one structure model, having a low loading B2 “In many cases, being fat is the result of a biological disorder” (0.0967), and B7 “Being fat is rarely caused by a lack of willpower” (-0.045). On the scree plot, we observed a clear elbow at the second component, suggesting that the first two components explained most of the variance and the additional components only contributed in a small proportion.

**Table 3:** Beliefs About Obese Persons scale: rotated component matrix. B1-B8 - items of the Beliefs About Obese Persons scale.

Item	Factor 1	Factor 2
B1	0.782	0.171
B2	0.088	-0.675
B3	0.841	0.004
B4	0.444	0.077
B5	0.751	-0.169
B6	0.899	-0.053
B7	-0.053	-0.697
B8	0.842	0.007

Reliability

Cronbach’s alpha for the AFA questionnaire was 0.862 (Dislike: 0.811; Fear of Fat: 0.867; Willpower: 0.860). The BAOP scale had a Cronbach’s alpha of 0.781 (the first component representing items B1, B3, B4, B5, B6, and B8 having an alpha value of 0.862, while items B2 and B7 of 0.642). The low value of Cronbach’s alpha of the second component indicates that we can use the BAOP scale with one factor (dimension), consisting of the following items: B1, B3, B4, B5, B6, and B8.

Evaluation of beliefs and attitudes against obesity in medical students

A weak but significant correlation was revealed between the students’ beliefs and attitudes against patients with obesity (Spearman’s rho = 0.380, p = 0.032). There were statistically significant differences (AFA: p = 0.02; BAOP: p = 0.03) between the scores of the students from the three medical faculties regarding attitudes toward individuals with obesity. Regarding the beliefs about persons with obesity, the BAOP scale produced a statistically significant difference between the students in Nursing and those in Medicine (p = 0.028) (Table 4). In addition, given that the AFA questionnaire consists of three subscales, namely, the stigma of obesity, willpower, and the fear of not gaining weight, we analyzed whether respondents from the three faculties differed in their perceptions of each AFA subscale. The results indicated no significant differences (p > 0.05) between the groups of the three specializations regarding the score that measures the fear of gaining weight. A significant difference was revealed regarding dislike (p = 0.016) between the students in nursing and medicine and between those in nutrition and medicine. Regarding the beliefs associated with the lack of willpower, a significant difference was noticed regarding the students in nutrition and medicine (p = 0.01) (Table 4).

**Table 4:** Evaluation of the questionnaire scores according to the faculty. *: Kruskal-Wallis; **: Dwass-Steel-Critchlow-Fligner pairwise comparisons. AFA = Anti-Fat Attitudes; BAOP = Beliefs About Obese Persons; SE = standard error

	Faculty	Faculty	Mean difference	SE	p*	p**
AFA	Nursing	Medicine	-9.72	3.85	0.02	0.048
		Nutrition	1.48	2.94		0.8
	Medicine	Nutrition	11.2	3.76		0.021
Dislike	Nursing	Medicine	-4.97	1.95	0.026	0.016
		Nutrition	-1.06	1.49		0.841
	Medicine	Nutrition	3.91	1.91		0.083
Fear	Nursing	Medicine	-2.889	1.59	0.125	0.205
		Nutrition	0.202	1.22		0.935
	Medicine	Nutrition	3.091	1.56		0.115
Willpower	Nursing	Medicine	-1.86	1.39	0.007	0.378
		Nutrition	2.33	1.06		0.091
	Medicine	Nutrition	4.2	1.35		0.01
BAOP	Nursing	Medicine	-1.737	0.837	0.03	0.028
		Nutrition	-0.958	0.64		0.216
	Medicine	Nutrition	0.779	0.818		0.41

## Discussion

Our study was conducted to evaluate the validity and reliability of two questionnaires assessing beliefs and attitudes against obesity in a sample of normal-weight students in medicine, nursing, and nutrition. Previous studies showed that normal-weight people were those who discriminated more against patients with obesity. However, the pandemic spread of obesity will expose healthcare professionals to relationships with people who are overweight or obese, and discriminatory beliefs and attitudes can hinder the professional role and undermine the empathic quality of medical care. The stigma associated with obesity stems from a failure to recognize obesity as a disease and its complexity. This inability to support individuals living with obesity violates the right of patients to receive and benefit from compassionate treatment. The prevalent narrative surrounding obesity focuses on the fact that the disease is the result of poor decisions, disregarding the numerous biological, psychological, social, commercial, and other contributing factors. Due to the failure to recognize obesity as a chronic, non-communicable disease, those living with obesity are stigmatized, even by healthcare professionals who are supposed to support them physically and emotionally. This is particularly crucial during the formative years when determining future convictions and professional conduct [30]. A previous study revealed that people subjected to a message stressing the lack of willpower as the primary cause of being overweight were more likely to share statements supporting this belief, while those exposed to a message focusing on the influence of heredity exhibited lower levels of weight controllability beliefs [31]. Greater confidence in clinical interactions with people with obesity was linked to experience and older age. Younger age was linked to greater dislike, fear, and conviction than those who are overweight or obese and lack willpower [32].

The first version of the AFA questionnaire was developed by Crandall [25] in 1994 on a sample of 251 undergraduate students in psychology, comparing the prejudice against individuals with obesity to racism. In our study, the principal factor analysis revealed three sections of the AFA questionnaire (Dislike, Fear of Fat, and Willpower). This is consistent with the original validation study, as well as with other studies conducted in Europe among the general population [21] and among social sciences students [33].

The BAOP scale was created by Allison et al. [23] in 1991 for graduate and undergraduate students as a one-factor structure. Few studies have examined the psychometric properties of the BAOP scale [33]. Like previous studies [20,34], we identified a single-factor structure. Items B2 and B7 had a weak correlation with the total score. Belonging to faculties with a medical profile could explain the lack of a strong relation to the total score of the B2 item (“In many cases, being fat is the result of a biological disorder”).

The reliability of the AFA questionnaire was 0.862 (Dislike: 0.811; Fear of Fat: 0.867; Willpower: 0.860). For the original scale, Cronbach’s alpha was 0.66 for the Willpower scale, 0.79 for the Fear of Fat scale, and 0.84 for the Dislike section [23]. In our study, the BAOP scale had moderate internal consistency (0.781). The Cronbach’s alpha calculated for the entire scale ranged between 0.61 [33] and 0.82 [25].

The study identified statistically significant variations in the attitudes and beliefs of students from three faculties (Nursing, Medicine, and Nutrition) toward individuals with obesity. However, these variations do not apply consistently to all areas examined. The analysis of beliefs regarding individuals with obesity showed a statistically significant distinction, particularly between students studying Nursing and Medicine, as indicated by the BAOP scale. A notable disparity in the attitudes toward obesity, as evaluated by the AFA questionnaire, was observed among Nursing and Medicine students, as well as between Nutrition and Medicine students (p = 0.016). The study found no statistically significant differences between the student groups of the three specializations in terms of their fear of gaining weight (p > 0.05), although attitudes regarding the stigma of obesity and the belief in lack of willpower exhibited considerable variations among certain faculty groups. A statistically significant difference was seen between Nutrition and Medicine students in their beliefs regarding the absence of determination (p = 0.01). The lack of significant differences in the fear of gaining weight across all specializations indicates a common apprehension or understanding about weight gain, which could be reflective of broader societal attitudes toward obesity.

Our study sample comprised students from three faculties that do not share the same curriculum and do not take the same number of nutrition classes. The curriculum of the Nursing Faculty includes one semester of nutrition, while nutrition is taught as an option class in the Medicine Faculty. Based on the attended faculty, notable distinctions were observed between students from the Medicine Faculty and those from the Nutrition Faculty. The participants from the former had a higher score in the Willpower section. Furthermore, a notable disparity was observed between nursing and medicine students in scores of the Dislike section, with nursing students exhibiting lower scores in this section. Higher scores on the Willpower subscale reflect the belief that being overweight is due to a personal lack of control in maintaining a healthy weight [10,16]. The BAOP scores differed significantly between Nutrition and Dietetics and General Medicine students.

The significant differences in perceptions of stigma and lack of willpower (specifically between Nursing and Medicine, and Nutrition and Medicine) could have profound implications. These perceptions could affect the approach of future healthcare professionals toward obesity management, potentially leading to different treatment strategies, patient counseling methods, and support mechanisms.

These findings emphasize the necessity for a more cohesive and thorough approach to obesity education in medical institutions. Addressing the differences in attitudes and beliefs to develop a comprehensive, sympathetic, and evidence-based understanding of obesity among future healthcare occupations would be mandatory to achieve high professionalism [35]. Integrating interdisciplinary learning experiences could mitigate these differences. Gaining insights from each other’s viewpoints cultivates a more homogeneous comprehension of obesity.

Although the study provides valuable insights, it is important to recognize its limitations, including the possible impact of uncontrolled cultural, regional, or institutional factors. Additional investigation could explore these aspects and evaluate the influence of targeted educational interventions on shaping students’ attitudes and beliefs toward obesity. In this study we focused specifically on healthcare students and, although it may be seen as a limitation, it highlights the importance of raising awareness on this aspect inside the healthcare system, at any level, due to the major impact of weight stigmatization of an important category of patients with complex comorbidities. First, the study participants were limited to one university, even if it was the largest and most important university of medicine in the northeast region of Romania. Extracting a convenience sample from a specific population (healthcare students) may represent a limitation, but it opens up the direction for future research on analyzing the impact of weight stigma on healthcare professionals (nurses, doctors, and healthcare technicians). Second, the test-retest reliability of the AFA and BAOP should be examined to assess the stability of the scales. However, the validity and reliability of the questionnaires were confirmed in our population. Third, the questionnaires assessed the level of obesity stigma in a sample of students with normal weight. The inclusion of students who are obese or overweight will be beneficial to compare the level of stigma according to the BMI category among medical students. Moreover, our sample did not include enough male students, which did not allow us to analyze the differences in stigma between the sexes. The questionnaires could also assist faculty members in comprehending the level of stigma among students and the significance of this factor in their relationships with patients.

## Conclusions

Our study confirms the validity and reliability of AFA and BAOP questionnaires in healthcare students. It highlights important differences in attitudes and beliefs regarding people with obesity, related to the knowledge background of respondents. The research highlights the complex and diverse views and attitudes toward obesity among medical students. It emphasizes the necessity for comprehensive educational practices and additional study to completely understand and tackle the different factors that impact these perceptions.

## References

[1] Popa S, Moţa M, Popa A (2016). Prevalence of overweight/obesity, abdominal obesity and metabolic syndrome and atypical cardiometabolic phenotypes in the adult Romanian population: PREDATORR study. J Endocrinol Invest.

[2] Alberga AS, Pickering BJ, Alix Hayden K (2016). Weight bias reduction in health professionals: a systematic review. Clin Obes.

[3] Kilbourne J (1994). Still killing us softly: advertising and the obsession with thinness. Feminist Perspectives on Eating Disorders.

[4] Spahlholz J, Baer N, König HH, Riedel-Heller SG, Luck-Sikorski C (2016). Obesity and discrimination - a systematic review and meta-analysis of observational studies. Obes Rev.

[5] Ryan L, Coyne R, Heary C (2023). Weight stigma experienced by patients with obesity in healthcare settings: a qualitative evidence synthesis. Obes Rev.

[6] Jones CA, Forhan M (2021). Addressing weight bias and stigma of obesity amongst physiotherapists. Physiother Theory Pract.

[7] Goff AJ, Lee Y, Tham KW (2023). Weight bias and stigma in healthcare professionals: a narrative review with a Singapore lens. Singapore Med J.

[8] Puhl RM, Latner JD, O'Brien K, Luedicke J, Danielsdottir S, Forhan M (2015). A multinational examination of weight bias: predictors of anti-fat attitudes across four countries. Int J Obes (Lond).

[9] Lawrence BJ, Kerr D, Pollard CM, Theophilus M, Alexander E, Haywood D, O'Connor M (2021). Weight bias among health care professionals: a systematic review and meta-analysis. Obesity (Silver Spring).

[10] Christenson A, Torgerson J, Hemmingsson E (2020). Attitudes and beliefs in Swedish midwives and obstetricians towards obesity and gestational weight management. BMC Pregnancy Childbirth.

[11] Arhire LI (2015). Orthorexia nervosa: the unhealthy obssesion for healthy food. Rev Med Chir.

[12] Sobczak K, Leoniuk K (2021). Attitudes of medical professionals towards discrimination of patients with obesity. Risk Manag Healthc Policy.

[13] Mihalache L, Graur LI, Popescu DS, Niţă O, Graur M (2012). Anthropometric parameters--predictive factors for cardio-metabolic diseases. Rev Med Chir.

[14] Poon MY, Tarrant M (2009). Obesity: attitudes of undergraduate student nurses and registered nurses. J Clin Nurs.

[15] Anguah KO, Christ SE (2024). Exposure to written content eliciting weight stigmatization: neural responses in appetitive and food reward regions. Obesity (Silver Spring).

[16] Jayawickrama RS, O'Connor M, Flint SW, Hemmingsson E, Lawrence BJ (2023). Explicit and implicit weight bias among health care students: a cross-sectional study of 39 Australian universities. EClinicalMedicine.

[17] Renold C, Deferm NP, Hauser R, Gerber P, Bueter M, Thalheimer A, Gero D (2023). The effect of a multifaceted intervention including classroom education and bariatric weight suit use on medical students' attitudes toward patients with obesity. Obes Facts.

[18] Talumaa B, Brown A, Batterham RL, Kalea AZ (2022). Effective strategies in ending weight stigma in healthcare. Obes Rev.

[19] Nutter S, Eggerichs LA, Nagpal TS (2024). Changing the global obesity narrative to recognize and reduce weight stigma: a position statement from the World Obesity Federation. Obes Rev.

[20] George TP, DeCristofaro C, Murphy PF (2019). Unconscious weight bias among nursing students: a descriptive study. Healthcare (Basel).

[21] Argyrides M, Anastasiades E, Charalambous Z, Michael K (2023). Validation of a Greek adaptation of the Anti-Fat Attitudes Questionnaire. Clin Obes.

[22] Rodat S (2020). Stigmata and the process of stigmatization: coping strategies and approaches to intervention for destigmatization. Anuarul Universitatii "Petre Andrei" Din Iasi - Fascicula: Drept, Stiinte Economice, Stiinte Politice.

[23] Allison DB, Basile VC, Yuker HE (1991). The measurement of attitudes toward and beliefs about obese persons. Int J Eat Disord.

[24] Kyriazos T (2018). Applied psychometrics: sample size and sample power considerations in factor analysis (EFA, CFA) and SEM in general. Psychology.

[25] Crandall CS (1994). Prejudice against fat people: ideology and self-interest. J Pers Soc Psychol.

[26] Taherdoost H (2016). Validity and reliability of the research Instrument; how to test the validation of a questionnaire/survey in a research. IJARM.

[27] Kaiser HF, Rice J (1974). Little Jiffy, Mark Iv. Educ Psychol Measure.

[28] Lance CE, Butts MM, Michels LC (2006). What did they really say?. Org Res Meth.

[29] Parmenter K, Wardle J (1999). Development of a general nutrition knowledge questionnaire for adults. Eur J Clin Nutr.

[30] Tomiyama AJ, Finch LE, Belsky AC, Buss J, Finley C, Schwartz MB, Daubenmier J (2015). Weight bias in 2001 versus 2013: contradictory attitudes among obesity researchers and health professionals. Obesity (Silver Spring).

[31] Ksinan AJ, Almenara CA, Vaculik M (2017). The effect of belief in weight controllability on anti-fat attitudes: an experimental manipulation. Eur Rev Appl Psychol.

[32] Phelan SM, Dovidio JF, Puhl RM (2014). Implicit and explicit weight bias in a national sample of 4,732 medical students: the medical student CHANGES study. Obesity (Silver Spring).

[33] Dedeli O, Bursalioglu SA, Deveci A (2014). Validity and reliability of the Turkish version of the attitudes toward obese persons scale and the beliefs about obese persons scale. Clin Nurs Stud.

[34] Tsai MC, Strong C, Latner JD, Lin YC, Pakpour AH, Lin CY, Wang SM (2019). Attitudes toward and beliefs about obese persons across Hong Kong and Taiwan: wording effects and measurement invariance. Health Qual Life Outcomes.

[35] Robinson J, Nitschke E, Tovar A, Mattar L, Gottesman K, Hamlett P, Rozga M (2023). Nutrition and physical activity interventions provided by nutrition and exercise practitioners for the general population: an evidence-based practice guideline from the Academy of Nutrition and Dietetics and American Council on Exercise. J Acad Nutr Diet.

